# Immunohistochemical staining of leptin is associated with grade, stage, lymph node involvement, recurrence, and hormone receptor phenotypes in breast cancer

**DOI:** 10.1186/s12905-017-0459-y

**Published:** 2017-11-09

**Authors:** Mohamad Nidal Khabaz, Amer Abdelrahman, Nadeem Butt, Lila Damnhory, Mohamed Elshal, Alia M. Aldahlawi, Swsan Ashoor, Basim Al-Maghrabi, Pauline Dobson, Barry Brown, Kaltoom Al-Sakkaf, Mohmmad Al-Qahtani, Jaudah Al-Maghrabi

**Affiliations:** 1 Department of Pathology, Rabigh Faculty of Medicine, King Abdulaziz University, Jeddah, Saudi Arabia; 2 Department of Family and Community Medicine, Rabigh Faculty of Medicine, King Abdulaziz University, Jeddah, Saudi Arabia; 3 Department of Medical Laboratory Technology, Faculty of Applied Medical Sciences, King Abdulaziz University, Jeddah, Saudi Arabia; 4 Department of Biological Sciences, Faculty of Science; Immunology Unit, KFMRC, King Abdulaziz University, Jeddah, Saudi Arabia; 5 Department of Radiology, King Abdulaziz University Hospital, Jeddah, Saudi Arabia; 6 Faculty of Medicine, King Abdulaziz University, Jeddah, Saudi Arabia; 7 Department of Human Metabolism, University of Sheffield, Sheffield, UK; 8 Center of Excellence in Genomic Medicine Research, King Abdulaziz University, Jeddah, Saudi Arabia; 9 Department of Pathology, Faculty of Medicine, King Abdulaziz University, P.O. Box 80205, Jeddah, 21589 Saudi Arabia

**Keywords:** Leptin, Breast cancer, Immunohistochemistry

## Abstract

**Background:**

Obesity is part of the established risk factors for breast cancer (BC) in postmenopausal females. Circulating leptin increases in parallel with the increase of body weight and fat reservoir.

**Methods:**

This research investigated the link between leptin phenotype and the clinicopathological factors in BC. A large set of breast cancer cases (449), and 27 non-cancerous tissue samples of breast were employed for leptin expression recognition using immunohistochemistry staining.

**Results:**

Cytoplasmic immunohistochemical staining of leptin was recognized in 376 (83.7%) and 25 (92.6%) of BC and control cases respectively. Leptin immunostaining were significantly associated with age, histotypes, grade, stage, lymph node involvement, tumor recurrence, hormone receptor phenotypes, ER and HER2 expressions, and *p*-values were (*P* = 0.0233), (*P* = 0.0001), (*P* = 0.050), (*P* = 0.0291), (*P* = 0.0300), (*P* = 0.0023), (*P* = 0.0021), (*P* = 0.0279) respectively. Reasonable proportion of cases with low staining score was more prevalent in all subgroups of clinicopathological parameters except ER- PR+ HER2- hormone receptor phenotype and mucinous carcinoma which showed high level of leptin immunoreactivity. Tumor recurrence is less prevailing in high score leptin immunostaining cases. Furthermore, Log Rank (Mantel-Cox) test findings revealed considerably different survival distributions were observed for the different categories of leptin immunostaining scores (*P* = 0.032). Negative leptin immunostaining is related to poor survival.

**Conclusions:**

Our preliminary findings support leptin clinical value in confirming BC diagnosis as well as prognosis. These results suggest that leptin molecule is an important biomarker that could identify type, grade, stage, lymph node involvement, relapse and prognosis in breast cancer.

## Background

Breast cancer (BC) is a shattering tumor and an important cause of worldwide death [1]. Recently published data stated that breast neoplasms are the most frequent malignancy among females with approximately 1,700,000 new registered cases and around 580,000 demises of BC in the United States of America in 2015 according to the American Cancer Society [2]. In Saudi Arabia, BC has a comparable rank among cancers and neoplasms accounting for (25.8%) of all registered neoplasms in females in 2012 as stated by the Saudi Cancer Registry [3].

BC has been distinguished as a high complex heterogeneous tumor with distinctive cellular origin and various histotypes, progression and metastatic potential [4]. Irrespective of noteworthy improvements in the diagnosis and treatment of BC, the tumor is still considered a big challenge to clinicians due to bad prognosis and big recurrence proportion in some histotypes of BC particularly triple negative, for instance up to 40% of newly registered cases relapse in 5 years [5]. The management of BC is subject to the clinicopathological parameters of patients, such as grade and stage of cancer as measures of pleasant or bad prognosis. Nevertheless, these factors are not enough to guess the clinical consequences and worse yet, may produce variations in a cluster of neoplasms with the same grade or stage. This is essentially due to heterogeneity of BC cells [6]. Therefore, it is necessary to find novel diagnostic markers and medicinal modalities which help in the diagnosis and prognosis of BC, enhance the stratification of high risk patients and improve clinical outcomes [7].

Leptin is one hundred and sixty seven amino acid residues molecule that is encrypted by the Obese gene (Ob) [8]. It was expressed firstly in white adipose tissue; however, later it was found that other tissues express leptin such as the liver, ovaries, placenta, stomach, pituitary gland and skeletal muscles [9]. It is now established that leptin has several roles and counted a member of adipokines [10]. Many investigations have illustrated the function of leptin in tumor cells proliferation, movement, invasion and apoptosis inhibition [11–13]. Some other reports have examined leptin function in several tumor development risks, but the results are controversial [14, 15]. Unquestionably definite proof is needed to elucidate leptin’s exact function in the growth and progress of breast tumors, as perception of leptin correlation with breast cancer can improve our awareness of breast carcinogenesis and support improving management and preventive plans. Thus, the current study describes leptin immunoexpression in BC and evaluates the association between leptin phenotype and the clinical factors as well as follow-up data of breast cancer.

## Methods

Four hundred fourty nine cases of BC and 27 control cases, which include fibroadenomas and normal breast tissue, were taken from the archive of pathological sciences department at King Abdulaziz University Hospital in Saudi Arabia. Sections from tumor paraffin blocks were hematoxylin and eosin stained and histologically evaluated. The unit of medical records provided us with patients’ clinicopathological data (age, size, type, grade and stage of tumors) (Table 1). WHO recommendation regarding grade and stage of BC was applied. All tumors and control cases blocks were utilized in the production of tissue microarray. This study has met all the instruction and requirement of the ethical committee approval.

**Table 1:** Describe the distribution of various clinicopathological variables with leptin immunostaining in breast cancer

		Leptin immunostaining						
		Negative		Low		High		
		Count	Row N %	Count	Row N %	Count	Row N %	*P*-Value
Type of tissue	Leptin in breast cancer	73	16.3%	274	61.0%	102	22.7%	0.0778
	Leptin in control group	2	7.4%	14	51.9%	11	40.7%	
Age in Years	<40	16	23.2%	30	43.5%	23	33.3%	0.0233
	40–49	16	14.0%	67	58.8%	31	27.2%	
	50–59	24	17.9%	89	66.4%	21	15.7%	
	60–69	11	16.2%	46	67.6%	11	16.2%	
	> = 70	6	11.8%	31	60.8%	14	27.5%	
	NA	0	0.0%	11	84.6%	2	15.4%	
Hormone receptor phenotype	ER- PR- HER2-	16	23.2%	36	52.2%	17	24.6%	0.0021
	ER- PR- HER2+	6	8.8%	40	58.8%	22	32.4%	
	ER- PR+ HER2-	0	0.0%	2	28.6%	5	71.4%	
	ER- PR+ HER2+	0	0.0%	7	77.8%	2	22.2%	
	ER+ PR- HER2-	7	14.0%	33	66.0%	10	20.0%	
	ER+ PR- HER2+	3	17.6%	11	64.7%	3	17.6%	
	ER+ PR+ HER2-	34	23.6%	81	56.3%	29	20.1%	
	ER+ PR+ HER2+	7	8.2%	64	75.3%	14	16.5%	
ER	ER-	22	14.4%	85	55.6%	46	30.1%	0.0279
	ER+	51	17.2%	189	63.9%	56	18.9%	
PR	PR-	32	15.7%	120	58.8%	52	25.5%	0.4410
	PR+	41	16.7%	154	62.9%	50	20.4%	
HER	HER2-	57	21.1%	152	56.3%	61	22.6%	0.0021
	HER2+	16	8.9%	122	68.2%	41	22.9%	
Lymph node involvement	NEGATIVE	33	17.8%	100	54.1%	52	28.1%	0.0300
	POSITIVE	40	15.2%	174	65.9%	50	18.9%	
Size of tumor	<2	10	16.7%	34	56.7%	16	26.7%	0.5784
	>5	13	12.0%	68	63.0%	27	25.0%	
	2–5	50	17.8%	172	61.2%	59	21.0%	
Grade	I	13	17.8%	42	57.5%	18	24.7%	0.0500
	II	45	18.8%	151	63.2%	43	18.0%	
	III	15	10.9%	81	59.1%	41	29.9%	
Histotype	DCIS	4	23.5%	9	52.9%	4	23.5%	0.0001
	Ductal	65	15.9%	255	62.2%	90	22.0%	
	Mucinous carcinoma	0	0.0%	1	11.1%	8	88.9%	
	Lobular	4	30.8%	9	69.2%	0	0.0%	
Stage	I	8	16.0%	27	54.0%	15	30.0%	0.0291
	II(a)	24	18.3%	69	52.7%	38	29.0%	
	II(b)	22	15.8%	92	66.2%	25	18.0%	
	III	4	6.1%	49	74.2%	13	19.7%	
	IV	15	23.8%	37	58.7%	11	17.5%	
Vascular Invasion	Negative	55	17.8%	184	59.5%	70	22.7%	0.4058
	Positive	18	12.9%	90	64.3%	32	22.9%	
Recurrence	No	57	14.3%	253	63.4%	89	22.3%	0.0023
	Yes	16	32.0%	21	42.0%	13	26.0%	

### Tissue microarray production (TMA)

Four hundred fourty nine cases of BC and 27 control cases were used to assemble tissue microarray [16]. TMA blocks have been cut and placed on coated slides, then they have been immunohistochemically stained.

### Immunohistochemistry staining protocol

Multimer molecule based scientific knowledge were employed in the immunohistochemistry staining of BC sections to apply anti-leptin rabbit polyclonal antibody with dilution ratio of 1 to 100 [catalog code: sc-842, Santa Cruz Biotechnology, USA), and ULTRAVIEW TM DAB visualizing protocol. Immunohistochemistry autostainer (BenchMark ULTRA, Ventana, Arizona, USA) was used for immunohistochemistry staining. Every staining run contained a slide treated with tris buffer in place of the Ob antibody as a negative control. Slide section of placenta tissue was employed as positive control. Cases with brown granular cytoplasmic stain in more than 5% of tumor cells were counted positive.

Leptin immunoreactivity has been scored, by two pathologists, for staining intensity and positively stained cells percentage. The frequency of positive cells was evaluated applying semiquantitative method in 3 fields with lenses of 40 amplification power. Leptin staining intensity has been given scores 0, 1, 2, 3 and 4 representing negative, weak, moderate and strong staining respectively. Scores of staining intensity has been presented as negative staining (0), low level immunoreactivity (1) and high level (2 and 3). When a disparity between the two pathologists’ staining scores has happened, the lowest score value was reported**.**

### Statistical analysis

All data were assessed statistically by IBM-SPSS software (version 21). All data values were presented as percentages and incidences. The association between clinicopathological factors of BC and leptin expression was explored statistically by chi-square test. Comparison of survival distributions for various leptin immunohistochemistry staining intensity levels was assessed applying Log Rank (Mantel-Cox) test in addition to Kaplan Meier survival curves. The level of significance was counted when *P* < 0.05.

## Results

All BC cases were reviewed and their clinicopathological factors have been presented in Table 1. The histotypes of breast cancer cases of the current study, in descending order, were infiltrating ductal carcinoma, ductal carcinoma in situ, infiltrating lobular carcinoma and mucinous carcinoma which counted 91.3%, 3.8%, 2.9 and 2% respectively (Table 1). The mean age of patients was 50.7 years varying from 24 to 94 years.

**Fig. 1:**
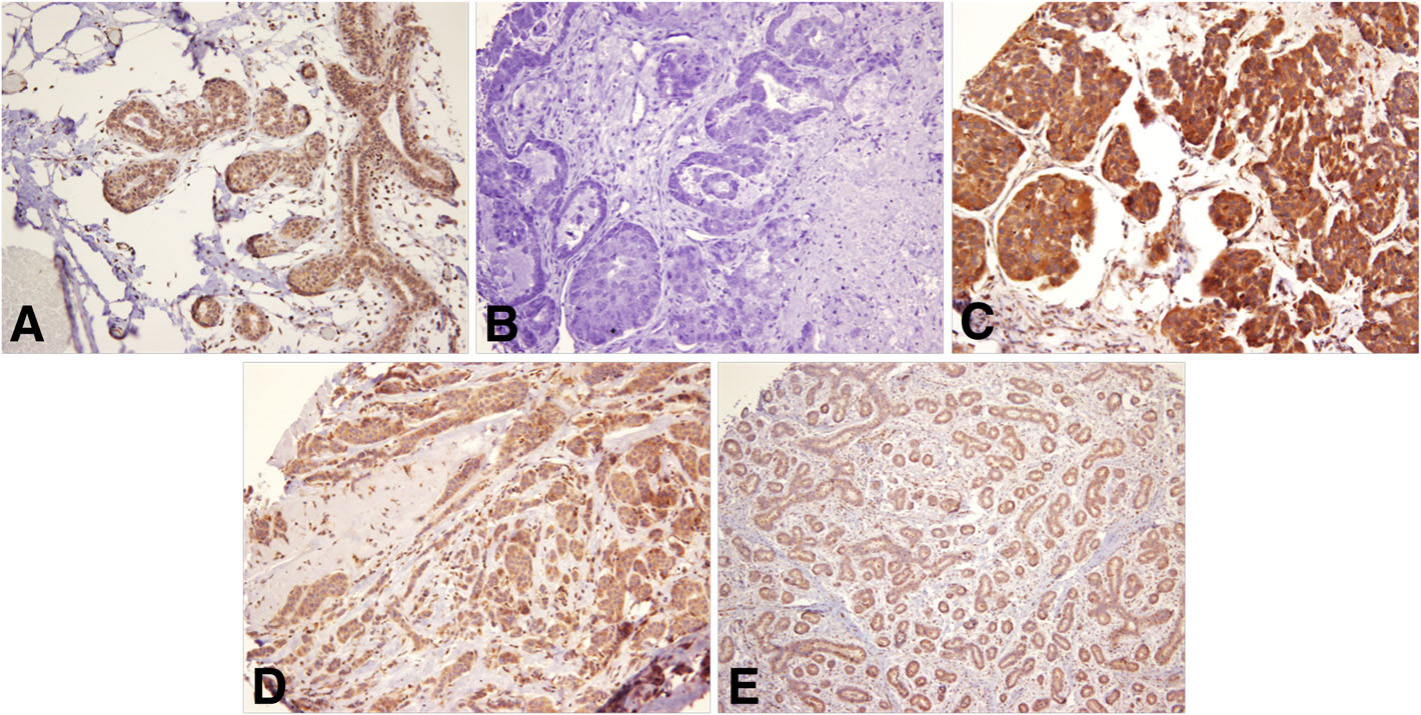
Granular cytoplasmic expression of leptin in breast cancer. **a** strong positive staining in normal breast tissue (20 X); **b** negative stained breast cancer (20 X); **c** strong positive staining in epithelial cells of breast cancer (20 X); **d** weak positive staining in epithelial cells of breast cancer (20 X); **e** weak positive staining in fibroadenoma (10 X)

Brown granular cytoplasmic leptin immunoexpression was detected in the transformed epithelium of 376 (83.7%) BC cases and 25 (92.6) cases of control group (Fig. 1).

Leptin expression did not show any statistical significant difference between BC and control cases. The distribution of leptin phenotypes which identified in BC transformed epithelial cells and its association with different clinicopathological variables were reported in Table 1. Percentage of positively stained cells ranged from 5% to 100% in breast tumors of the present study. About 40% of breast cancer cases showed leptin immunoreactivity in more than 50% of their transformed epithelial cells. Small fraction of cases (<10%) showed moderate to strong leptin immunoreactivity in stromal cells; however, these cases were of no statistical significance.

**Fig. 2:**
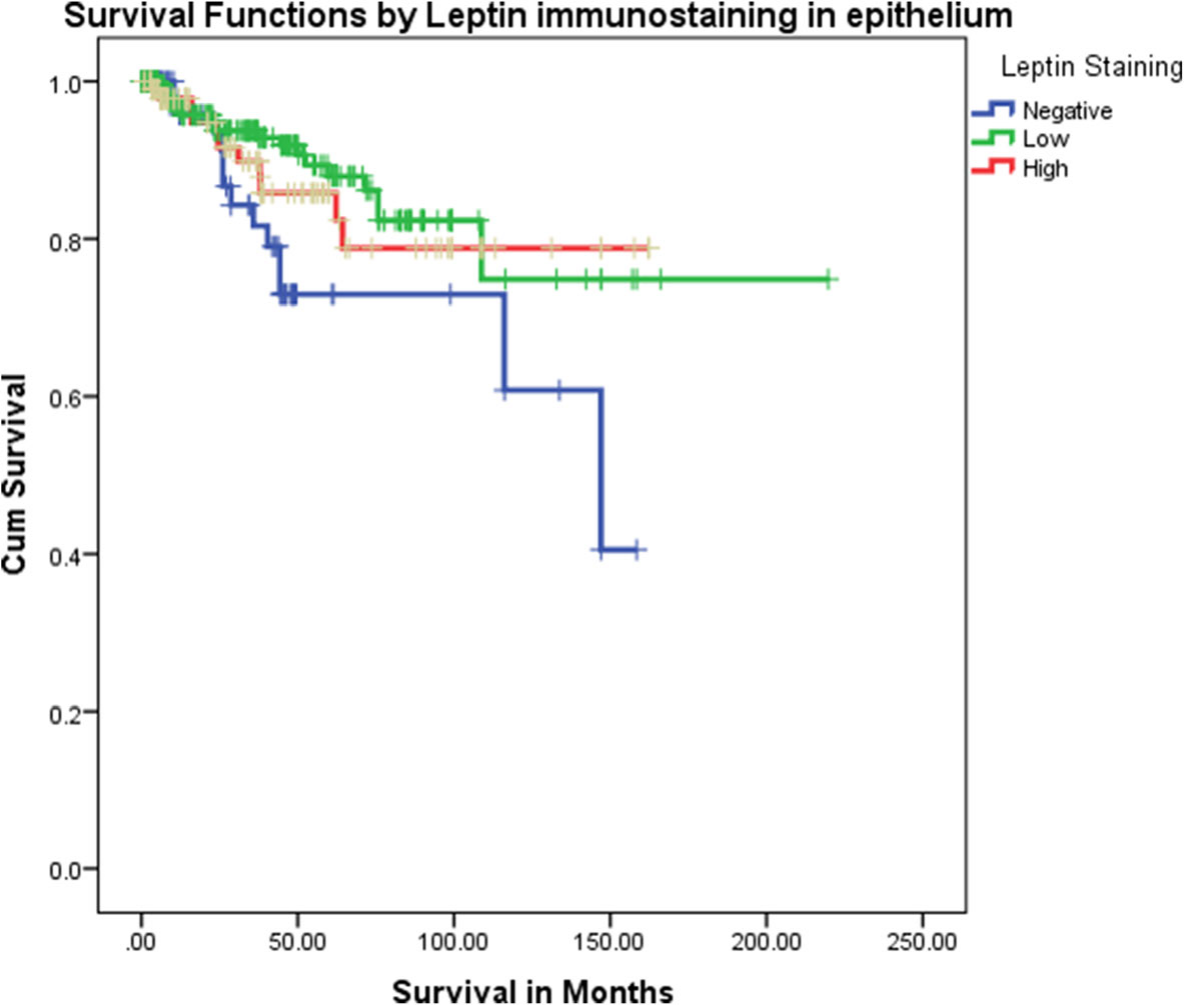
Kaplan Meier survival curves by pattern of leptin immunostaining shows significantly poor survival behavior associated with negative leptin immunostaining in breast cancer

Leptin immunostaining is significantly related with age (*P* = 0.0233), reasonable proportion of low scores staining is observed in all age groups. Breast cancer histotypes showed significant association with leptin immunostaining (*P* = 0.0001). DCIS, invasive ductal carcinoma and invasive lobular carcinoma histotypes showed more frequently low scores of leptin immunostaining while the vast majority of mucinous carcinomas were of high immunostaining scores. Grade of breast tumors is marginally significant with leptin immunostaining (*P* = 0.050). Grade II is more frequent with low leptin immunoreactivity. Breast carcinoma stage was also significantly associated with leptin expression (*P* = 0.0291). A considerable fraction of stage II (b) and stage III were found to be common with low leptin immunostaining. Significantly, more cases with metastases in lymph nodes were observed in low score staining (*P* = 0.0300). Tumor recurrence was significantly associated with cases of low leptin immunostaining scores (*P* = 0.0023). Recurrence is less prevailing in cases with high score of leptin immunostaining. Furthermore, hormone receptor phenotypes were significantly associated with leptin expression (*P* = 0.0021). All hormone receptor phenotypes were significantly more prevalent in cases with low staining scores except “ER- PR+ HER2- “which was more common in cases with high leptin scores. Distributions of ER and HER2 expression were significantly different by leptin immunostaining (*P* = 0.0279 and P = 0.0021 respectively), while PR expression was not. Log Rank (Mantel-Cox) test outcomes revealed that significant different survival distributions were observed for different categories of leptin immunostaining scores (*P* = 0.032). Negative leptin immunostaining is related to poor survival significantly (Fig. 2). No significant associations of leptin immunostaining in transformed epithelium with tumor size, vascular invasion and type of tissue (malignant vs control) were observed.

## Discussion

Several serological studies stated evidences that elevated leptin concentration in serum is correlated with breast cancer risk and counted it as an independent risk factor, in addition to its involvement in many malignancy stages including as cell growth, invasion, migration, metastases, recurrence and therapy response in some organs such as liver [17], lung [18], stomach [19], thyroid [20], uterus [12], colon [21]. A number of investigations were launched to identify the mechanisms which link leptin with tumor growth and progression of breast cancer [22, 23]. Some studies reported a direct role of leptin in BC development and aggression, and others showed that serum adipocytokines apply their biological roles on recipient tissues and cells not just by typical endocrinological mechanisms but additionally via autocrine or paracrine systems [11, 22–29]. However, leptin expression in mammary tumor tissue is not characteristic of blood leptin levels, but could be a result of the paracrine mechanism [22]. Furthermore, leptin intervenes estrogen effects on malignant tissue via a paracrine pathway, as well as enhances other influences that participate in cell growth and angiogenesis during breast cancer development [23, 30]. Moreover, leptin autoregulation enhances its signal through motivating its expression and its receptor, thus supports an autocrine mechanism [29]. To the best of our knowledge, few studies evaluated leptin expression in breast cancer tissues (Table 2) [31–40] of which the outcomes failed to confirm the results of leptin serological studies and the correlation of leptin immunoexpression with clinicopathological findings of breast carcinoma patients.

**Table 2:** Correlation between high level of leptin immunoreactivity and clinicopathological parameters in the current study compared to previous studies

Previous studies	Leptin immunostaining prevalence in breast cancer cases	Leptin immunostaining prevalence in noncancerous breast tissue	Age	Size of tumor	Histotype	Grade	Stage	Recurrence	Lymph Node involvement	Hormone receptor phenotype (ER, PR, HER2)	ER expression	PR expression	HER2 expression	Vascular Invasion	Alive/Deceased status	Survival
The current study	83.7% (61% weak & 22.7% strong)	92.6% (51.9% weak & 40.7% strong)	P = 0.0233	NS	P = 0.0001	All grades low scores *P* = 0.0500	P = 0.0291	P = 0.0023	P = 0.0300	ER-, PR+, HER2-P = 0.0021	ER+ P = 0.0279	NS	P = 0.0021	NS	NS	Absent or Weak staining -Poor survival
[1]	100% (7.9% weak & 92.1% strong)	100% (100% weak)	NS	NS	NS		NS		NS		NS	NS				Overexpression – poor survival
[2]	60%	0%														
[3]	86.4% (30.4% weak & 56% strong)	43.3% (43.3% weak)	NS	NS		High grade high scores *P* = 0.031			NS		NS					
[4]							NS				NS	NS	NS			NS
[5]	79.6% (52.5% weak & 27.1& strong)	77.5% (50% weak & 27.5% strong)														
[6]	79.6%			NS	NS	NS	NS		NS		NS	NS	NS	NS		
[7]	85%	76.5%		NS							NS	NS				
[8]	39%		NS			NS	NS			ER-, PR-, HER2-*P* = 0.022	NS	NS	NS			NS
[9]	83%			NS	*P* < 0.001	NS			NS		NS	NS	NS	NS		
[10]	61%	40%									NS	NS	NS			

*NS* not significant

In our report, the incidence of leptin immunostaining (92.6%) in the 27 control cases, which was seen only in the cytoplasmic space of glandular epithelial cells, is almost similar to the results of Ishikawa, Kitayama and Nagawa [31] who described positive leptin immunohistochemistry staining in 100% of noncancerous breast tissue, and higher than those of Caldefie-Chezet and associates [32], Garofalo et al. [33], Jarde and coworkers [35], and Colbert and colleagues [40]. In respect of the percentage of positive breast carcinoma cases for leptin immunoexpression, our results are in line with those of Garofalo and associates [33], Fiorio and coworkers [36], Jarde and associates [37] and Jeong team [39] who detected leptin immunoexpression in 86.4%, 79.6%, 79.6% and 83% of breast carcinomas respectively, but with different immunoreactivity levels; and varied from those of Ishikawa, Kitayama and Nagawa [31], Caldefie-Chezet and associates [32], Kim [38] and Colbert and colleagues [40].

Our investigation is pioneer to report immunohistochemical staining of leptin is considerably correlated with patients’ clinicopathological findings such as age, histotype, grade, stage, recurrence, lymph node involvement, hormone receptor phenotype, ER expression, HER2 expression and survival of patients with breast carcinoma. Whereas, all the previous studies (Table 2) did not detect similar correlation except Ishikawa, Kitayama and Nagawa [31] who reported that strong leptin immunostaining is only associated with poor survival; Garofalo and associates [33] associated leptin immunostaining only with high grade tumors; Jeong and colleagues [39] linked leptin expression with histotype of breast cancer; and Colbert and coworkers [40] stated significant relationship with triple negative breast carcinoma.

Nevertheless, our results are in agreement with several other reports which have documented that immunoexpression of leptin is linked with one or more of the clinical factors such as tumor stage, infiltration, metastasis, relapse, therapy resistance and bad prognostic outcomes of several tumors including laryngeal cancer [41], esophageal cancer [42], stomach cancer [43], lung cancer [44], and thyroid cancer [45].

Main differences between our report and previous ones can be justified by techniques sensitivity, the diversity of populations, variations in sample size and the semi-quantitative reading of immunostaining. Still, studies with broader panel of cases are certainly of great value for assessing the value of leptin immunostaining in diagnoses and prognoses of breast malignancies.

## Conclusions

Leptin immunostaining is a useful method in supporting the diagnoses and prognoses of breast carcinoma. Our findings proposes that leptin could be a helpful biomarker in identifying the histotype, stage, grade, relapse and prognosis in BC. The association of leptin immunostaining with many clinicopathological factors proposes a role of leptin in BC progression.

## Abbreviations

BC: Breast cancer
DCIS: Invasive ductal carcinoma
ER: Estrogen receptor
HER2: Human epidermal growth factor receptor 2
Ob: Obese gene
PR: Progesterone receptor
TMA: Tissue microarray
WHO: World Health Organization
